# High-Quality Draft Genome Sequences of Two Deltaproteobacterial Endosymbionts, Delta1a and Delta1b, from the Uncultured Sva0081 Clade, Assembled from Metagenomes of the Gutless Marine Worm Olavius algarvensis

**DOI:** 10.1128/MRA.00276-20

**Published:** 2020-04-16

**Authors:** Yui Sato, Juliane Wippler, Cecilia Wentrup, Nicole Dubilier, Manuel Kleiner

**Affiliations:** a Max-Planck Institute for Marine Microbiology, Department of Symbiosis, Bremen, Germany; b University of Vienna, Department of Microbiology and Ecosystem Science, Vienna, Austria; c University of Bremen, Faculty 2 Biology/Chemistry, Bremen, Germany; d North Carolina State University, Department of Plant & Microbial Biology, Raleigh, North Carolina, USA; Georgia Institute of Technology

## Abstract

Here, we present high-quality metagenome-assembled genome sequences of two closely related deltaproteobacterial endosymbionts from the gutless marine worm Olavius algarvensis (Annelida). The first is an improved draft genome sequence of the previously described sulfate-reducing symbiont Delta1. The second is from a closely related, recently discovered symbiont of *O. algarvensis*.

## ANNOUNCEMENT

Olavius algarvensis lacks a digestive and excretory system and lives in obligate association with bacterial endosymbionts. These symbionts provide the host with nutrition through carbon fixation and recycle its waste products (1–4). The described symbionts of *O. algarvensis* are two Deltaproteobacteria (Delta1 and Delta4) and two *Gammaproteobacteria* (Gamma1 [“*Candidatus* Thiosymbion algarvensis”] and Gamma3 [5]), whose draft genome sequences have been published (1), as well as a spirochete. We report an improved draft genome sequence of the Delta1 symbiont and a draft genome sequence of a newly discovered deltaproteobacterial symbiont, closely related to the Delta1 symbiont. Due to their close phylogenetic relationship, we named these symbionts Delta1a (formerly Delta1) and Delta1b. Both symbionts belong to the widespread Sva0081 clade of uncultured, free-living deltaproteobacterial sulfate reducers (6).

*O. algarvensis* specimens were collected in Sant’Andrea (Elba, Italy; 42°48′26.00″N, 10° 8′28.00″E) in June 2014 as described previously (3), and were stored in RNAlater (Ambion Life Technologies) at 4°C. DNA was individually extracted from 2 worms using the AllPrep DNA/RNA kit (Qiagen). Two separate Illumina TruSeq DNA libraries were prepared at the Max Planck Institute for Plant Breeding Genome Center and sequenced on the Illumina HiSeq 2000 platform (2 × 100 bp), resulting in 28,259,255 and 33,422,854 raw read pairs from Delta1a and Delta1b libraries, respectively. Raw reads were quality controlled using bbduk (minimum [min.] kmer, 11; min. length, 36 bp; min. phred quality, 2) (BBMap toolkit v36.86; https://sourceforge.net/projects/bbmap/) and BayesHammer implemented in SPAdes v3.9.1 (7, 8). Clean reads (26,440,546 and 31,821,934 sequence pairs for Delta1a and Delta1b libraries, respectively) were *de novo* assembled with MEGAHIT v1.0.6 (preset, –meta) (9). Draft symbiont bins were obtained using MetaBAT v0.26.3 (10) and were further refined using Bandage v0.08.1 (11). Completeness and contamination were estimated with CheckM v1.0.5 (12). Enveomics tools (13) was used to calculate average nucleotide and amino acid identities. Genome statistics were calculated with QUAST v4.6.1 (14). Genome contents were annotated and analyzed using RASTtk (15–17) and Pathway Tools v21.0 (18). Default parameters were used for all software unless otherwise noted.

Two metagenome-assembled genomes (MAGs) were identified as Delta1a and Delta1b based on their 16S rRNA sequences. Delta1a contains the identical 16S rRNA sequence as that contained by Delta1 from the prior study (1). Delta1a and Delta1b share 98.8% identical 16S rRNA sequences, while their average nucleotide and amino acid identities of 82.0% and 78.1%, respectively, clearly separate them as individual species. The Delta1a draft genome has 10.62 Mb (*N*_50_, 10.5 kb; G+C content, 49.9%) and greater completeness (96.0%), more gene features (13,141 protein coding sequences [CDSs]), and only slightly increased contamination (2.4%) than the former Delta1 draft genome (11.22 Mb, 83.7% completeness, 10,680 CDSs, and 1.6% contamination). The Delta1b draft genome has 9.89 Mb (*N*_50_, 44.4 kb; G+C content, 48.2%), 96.0% completeness, and 0.8% contamination and contains 10,871 CDSs. Both MAGs conform to the Genomic Standards Consortium Minimum Information about a Metagenome-Assembled Genome (GSC MIMAG) standard for high-quality draft genomes (19). The functions carried by their genomes indicate that both symbionts are sulfate reducers capable of carbon monoxide and hydrogen oxidation. Both encode various transporters for the uptake of waste compounds produced by the host, such as short-chain fatty acids, urea, and glycine betaine, highlighting their proposed role in host waste disposal and recycling.

### Data availability.

The MAGs and raw sequences of Delta1a and Delta1b endosymbionts have been deposited in the European Nucleotide Archive under accession no. PRJEB28157, using the data brokerage service of the German Federation for Biological Data (GFBio) (20), in compliance with the Minimal Information about Any (x) Sequence (MIxS) standard (21).
